# Effects of pesticide exposure on oxidative stress and DNA methylation urinary biomarkers in Czech adults and children from the CELSPAC-SPECIMEn cohort

**DOI:** 10.1016/j.envres.2023.115368

**Published:** 2023-04-01

**Authors:** Tomáš Janoš, Ilse Ottenbros, Lucie Bláhová, Petr Šenk, Libor Šulc, Nina Pálešová, Jessica Sheardová, Jelle Vlaanderen, Pavel Čupr

**Affiliations:** a RECETOX, Faculty of Science, Masaryk University, Kotlarska 2, Brno, Czech Republic; b Institute for Risk Assessment Sciences, Utrecht University, Utrecht, the Netherlands; c Center for Sustainability, Environment and Health, National Institute for Public Health and the Environment (RIVM), Bilthoven, Netherlands

**Keywords:** Epigenetics, DNA methylation, Oxidative stress, Current-use pesticides (CUPs), Urine

## Abstract

Current-use pesticide (CUP) exposure occurs mainly through diet and environmental application in both agricultural and urban settings. While pesticide exposure has been associated with many adverse health outcomes, the intermediary molecular mechanisms are still not completely elucidated. Among others, their roles in epigenetics (DNA methylation) and DNA damage due to oxidative stress are presumed. Scientific evidence on urinary biomarkers of such body response in general population is limited, especially in children.

A total of 440 urine samples (n = 110 parent-child pairs) were collected during the winter and summer seasons in order to describe levels of overall DNA methylation (5-mC, 5-mdC, 5-hmdC, 7-mG, 3-mA) and oxidative stress (8-OHdG) biomarkers and investigate their possible associations with metabolites of pyrethroids (3-PBA, t/c-DCCA), chlorpyrifos (TCPY), and tebuconazole (TEB-OH). Linear mixed-effects models accounting for intraindividual and intrahousehold correlations were utilized. We applied false discovery rate procedure to account for multiplicity and adjusted for potential confounding variables.

Higher urinary levels of most biological response biomarkers were measured in winter samples. In adjusted repeated measures models, interquartile range (IQR) increases in pyrethroid metabolites were associated with higher oxidative stress. t/c-DCCA and TCPY were associated with higher urinary levels of cytosine methylation biomarkers (5-mC and/or 5-mdC). The most robust association was observed for tebuconazole metabolite with 3-mA (−15.1% change per IQR increase, 95% CI = −23.6, −5.69) suggesting a role of this pesticide in reduced demethylation processes through possible DNA glycosylase inhibition.

Our results indicate an urgent need to extend the range of analyzed environmental chemicals such as azole pesticides (e.g. prothioconazole) in human biomonitoring studies. This is the first study to report urinary DNA methylation biomarkers in children and associations between CUP metabolites and a comprehensive set of biomarkers including methylated and oxidized DNA alterations. Observed associations warrant further large-scale research of these biomarkers and environmental pollutants including CUPs.

## Highlights

• We examined the associations of CUPs with DNA methylation and oxidation biomarkers.
• Higher levels of most urinary response biomarkers were measured in winter season.
• Pyrethroid metabolites were associated with oxidative stress biomarker.
• All assessed CUPs were associated with changes in DNA methylation patterns.
• Possible mode of azole pesticides action via DNA glycosylase inhibition was proposed.

## 1 Introduction

Pesticides are agrochemicals used worldwide for the protection of crops from various types of pests, as well as to control detrimental organisms (e.g., rodents) or vector-borne diseases. Their role is key in sufficient food production and management of human diseases. However, their overproduction and overuse are problematic not only in agricultural areas but in urban environment as well (Md Meftaul et al., 2020). Although some progress in recent years toward safer use of pesticides was achieved, current-use pesticides (CUPs) still represent a potential risk for human health (K. H. Kim et al., 2017). Exposure to CUP mixtures can occur through several routes and pathways. While diet has been identified as the main exposure route (Becker et al., 2006; Nougadère et al., 2012), non-dietary routes such as direct skin contact, exposure via house dust, providing a long-term residential exposure route or airborne pesticides inhalation play additionally a significant role, especially when considering population living close to agricultural lands (K. H. Kim et al., 2017; Dereumeaux et al., 2020).

To better understand health consequences and provide risk assessment, CUPs or their metabolites are monitored in human fluids such as serum (Chang et al., 2017) or urine (Dereumeaux et al., 2018). As collection of urine samples is less invasive, urine is available in adequate quantity and easily collected by the study participants themselves, urine collection is often the preferred method over blood samples (Oerlemans et al., 2021).

Several previously conducted human biomonitoring studies suggested an association between CUP exposure and potential adverse health effects, for instance male reproductive effects (sperm quality and sperm DNA damage, reproductive hormone disorders), neurobehavioral development problems, endocrine disrupting effects, or even cancer (Koureas et al., 2012; González-Alzaga et al., 2014; Saillenfait et al., 2015). Moreover, specific population sub-groups (e.g. children, pregnant women) could be more sensitive to the pesticide exposure than others (K. H. Kim et al., 2017).

While exposure has been associated with many above mentioned health outcomes, a majority of the intermediary molecular mechanisms by which CUPs could exert their harmful effects are still not completely elucidated. Among various mechanisms linked to pesticide-induced chronic diseases, their roles in epigenetics (DNA methylation) and oxidative DNA damage are presumed (Banerjee et al., 2001; Collotta et al., 2013; Sabarwal et al., 2018). Both methylated and oxidized DNA lesions have been previously associated with various health outcomes, such as cancer (Robertson, 2005; Guo et al., 2017), pulmonary (Neofytou et al., 2012) and cardiovascular (di Minno et al., 2016) diseases, male reproductive issues (Pan et al., 2016) or neurodegenerative disorders (Guo et al., 2017; Gątarek et al., 2020). DNA methylation is one of the most important epigenetic modifications with the ability to regulate gene expression, cellular differentiation and genetic imprinting (Gehring et al., 2009). It comprises adding a methyl group enzymatically, typically but not exclusively to the cytosine nucleotide (DNA methylation) as well as nonenzymatically to the adenine and guanine nucleotide (methylated DNA lesions) (Hu et al., 2012). Abnormal methylation levels could be caused by both methylation and demethylation processes (Chen and Riggs, 2011). Furthermore, DNA may be altered by the hydroxyl radical, hazardous reactive oxygen species (ROS) with subsequent methylated and oxidized DNA lesions creation. ROS also play a role in the oxidation of methionine which could contribute to the formation of methyl radicals, leading to potential chemical DNA hypermethylation (Hu et al., 2012). Under normal circumstances, there is a balance between ROS production and antioxidant activity or accumulation. If a disbalance occurs, ROS overproduction can cause oxidative damage to nucleic acids, including both nuclear and mitochondrial DNA and RNA, with abundant and stable adduct 8-hydroxy-2′-deoxyguanosine (8-OHdG) creation (Valavanidis et al., 2009). Both methylated and oxidized DNA alterations could be removed by several pathways, especially base excision repair (BER), nucleotide excision repair (NER), oxidation or hydrolysis and their resulting products appear in the bloodstream and are excreted and present in urine (Hu et al., 2011, 2012; Fleming et al., 2015). These biomarkers then reflect the DNA repair processes in the whole body and have been previously detected in urine and proposed as possible response biomarkers to exogenous exposures (Valavanidis et al., 2009; Hu et al., 2011, 2012; Pan et al., 2016; Graille et al., 2020) or promising early biomarkers of several diseases (Pan et al., 2016; Onishi et al., 2019). Furthermore, some of them were associated with exposure to other environmental pollutants such as phthalates, benzene or organophosphate flame retardants (Pan et al., 2016; Ait Bamai et al., 2019; Lovreglio et al., 2020). Nevertheless, there is no study investigating associations of urinary response biomarkers of both oxidized and methylated DNA alterations in relation to CUP exposure in general population. Moreover, there is a complete knowledge gap in urinary DNA methylation biomarkers in children.

Therefore, in the present study we (I.) determined and described urinary levels of DNA methylation and oxidative stress biomarkers in samples from winter and summer seasons among Czech adults and children from the CELSPAC-SPECIMEn cohort and (II.) investigated possible associations between response biomarkers (DNA methylation and oxidative stress) and urinary levels of CUP metabolites or parental compounds.

## 2 Materials and methods

### 2.1 Study population and sample collection

The present study is part of the SPECIMEn (Survey on PEstiCIde Mixtures in Europe) study with the initial aim to assess exposure to pesticide mixtures in the general population (Ottenbros et al., 2023). This work is focused on the Czech cohort of the SPECIMEn study: CELSPAC-SPECIMEn (Central European Longitudinal Studies of Parents and Children). The CELSPAC-SPECIMEn study in the Czech Republic received ethical approval under ref. no. ELSPAC/EK/3/2019. A detailed description of the study protocol has been published previously (Šulc et al., 2022). Briefly, adult-child pairs were recruited during 2019 and 2020. Only adults older than 20 years with school-age (5–12 years) children were accepted into the study. Farmers and other professionals with potential occupational exposure to CUPs were excluded. Urine sample collection took place in two rounds, from mid-January 2020 to mid-March 2020 (hereinafter “winter season”) and from the end of May 2020 to the end of July 2020 (hereinafter “summer season”). Samples were not collected on weekends and Mondays due to possible differences in the participant behavior during the weekend. Each participant received the materials needed for urine collection, including urine containers, collection cups, storage bags, informed consent, and a questionnaire. Urine samples (first-morning void) were self-collected by participants then stored in the fridge until the arrival of the field worker. Samples were transported to the laboratory under refrigeration, aliquoted, and stored at −80 °C until analysis. The whole process from sample collection to sample storage took no longer than 24 h. One adult-child pair was excluded from further analyses because of the dropout during the study course. The final number of participants was 110 adults and 110 children sampled in two seasons (total n = 440).

### 2.2 Urinary biomarkers

Collected urine samples (n = 440) were analyzed for twelve biomarkers of exposure to CUPs and six response biomarkers. The selection of CUP biomarkers was based on the recommendation of HBM4EU (The European Human Biomonitoring Initiative) (Prioritized substance group: Pesticides) (Ougier et al., 2021), the annual reports of Plant Protection Products in the Czech Republic (CISTA, 2022), and on the European Food Safety Authority (EFSA) report (EFSA, 2021). Urinary CUP metabolite concentrations were measured by means of high-performance liquid chromatography (HPLC) in tandem with a mass spectrometer-mass spectrometer system (MS-MS). Detailed description of the method, quality assurance and quality control, coupled with the list of exposure biomarkers have been described elsewhere (Šulc et al., 2022). Only biomarkers with detection frequency at least 40% of all the samples were included in the current study: 3-phenoxybenzoic acid (3-PBA) and trans/cis-3-(2,2-dichlorovinyl)-2,2-dimethylcyclopro-pane carboxylic acid (t/c-DCCA) as pyrethroid metabolites, chlorpyrifos metabolite 3,5,6-trichloro-2- pyridinol (TCPY) and tebuconazole metabolite hydroxy-1-tebuconazole (TEB-OH).

Response biomarkers (biomarker of oxidative stress and DNA methylation) were selected based on prior literature research and consist of (I.) urinary biomarker of oxidative damage, specifically 8-hydroxydeoxyguanosine (8-OHdG) and (II.) biomarkers of potential nucleic acid methylation, namely 5-methylcytosine (5-mC), its deoxynucleoside and oxidized modification: 5-methyl-2′-deoxycytidine (5-mdC) and 5-hydroxymethyl-2′-deoxycytidine (5-hmdC); 7-methylguanine (7-mG) and 3-methyladenine (3-mA). Extraction of selected epigenetic biomarkers and biomarker of oxidative stress was performed according to the previously published study (Bláhová et al., 2023 – manuscript submitted). In short, the urine sample were thawed and 10 μL of internal standards mixture was added to 0.5 mL of each sample and calibration solutions (0, 0.05, 0.5, 5, 50, 500 μg/L in 0.1% v/v formic acid). Samples were freeze-dried and then extracted with isopropanol. Insoluble particles were removed by centrifugation, supernatants were evaporated to dryness and further redissolved in 0.1% formic acid (v/v). Possible residual particles were removed using microspin filters (0.2 μm; cellulose acetate; Fisher Scientific). Filtrates were stored in glass inserts at −20° until the analyses.

The analysis of selected response biomarkers was done by ultra-performance liquid chromatograph Acquity UPLC (Waters, Ireland) followed by tandem mass spectrometer Xevo TQ-S (Waters, Ireland). The mobile phase consisted of 0.1% formic acid in water (A) and acetonitrile acidified by 0.1% formic acid (B). The binary pump gradient was linear (3% B to 80% B at 5 min). The flow rate was 0.2 mL/min, and 10 μL of the individual sample was injected for the analysis. Analytes were detected in ESI positive ion mode and the ionization parameters were as follows: capillary voltage, 2.5 kV; the source temperature and the desolvation temperature, 150 and 750 °C, respectively; the cone gas flow, 150 (L/h); the cone voltages, 30 V; the desolvation gas flow, 750 (L/h); and the collision gas flow, 0.15 mL/min. The concentrations of response biomarkers in extracts were determined from a calibration curve with the use of an internal standard (software Mass Lynx, Manchester, UK). Concentrations of 5-mC, 5-mdC and 5-hmdC were corrected for the content of internal standard 5-mdC d3; the concentration of 3-mA was corrected for the content of internal standard 3-mA d3 and concentrations of 7-mG and 8-OHdG were corrected for the content of internal standard 8-OHdG 15N5. Quality assurance and quality control samples, including blanks, spiked samples (5.0 ng/mL of all analytes in 0.1% formic acid) and model urine samples (in house reference material with known level of biomarkers) were repeatedly extracted and included in the analysis. Quality control samples were analyzed after every 25 urine samples and found repeatability was acceptable (relative standard deviation (RSD) ≤ 15%). Five procedural blanks were analyzed in each analytical run with concentration below LOD. Mass spectrometer and other validation parameters are listed in SI Table 1.

### 2.3 Data analysis

Data were analyzed and visualized in the R programming language, version 4.1.1 (R Core Team, 2021). All urinary biomarkers were corrected for urine dilution using specific gravity (SG). SG was measured at the time of response biomarkers analysis using handheld refractometer Atago PAL-10 S, Japan. SG-corrected concentrations were created using following formula:

$$B_{c}=B\times \left(\frac{{SG}_{avg.}-1}{SG-1}\right)$$

where B_C_ is the SG corrected concentration of a biomarker, B is the measured concentration of a biomarker, SG_avg._ is the average specific gravity of all adult (1.017) or child (1.021) samples and SG is the specific gravity of the respective urine sample (Sauvé et al., 2015). Values below limit of quantification (LOQ) and/or limit of detection (LOD) were imputed on the basis of maximum likelihood multiple estimation dependent on observed values, which were expected to have a lognormal distribution (Lubin et al., 2004). The imputation was done only for compounds detected in at least 40% of all the samples. Before statistical analyses, we used natural log (ln) transformation to achieve normal distribution of measured biomarkers. The Pearson coefficient (r) was used to determine correlations between response biomarkers.

To examine the associations of response biomarkers with CUP metabolites, the linear mixed effect (LME) model was utilized. Random intercepts for participant ID and specific household were used to account for intraindividual and intrahousehold correlations. For modeling, uncorrected concentrations were used and models were adjusted for specific gravity of urine as proposed by (Barr et al., 2005). We first constructed basic model with specific gravity (continuous), age (in years, continuous), sex (male, female) and body mass index (BMI) (in kg/m^2^, continuous) adjustment and then potential variables were added to examine the associations. Minimal sufficient adjustment set of covariates included in LME models were selected based on prior knowledge and direct acyclic graphs (DAGs) approach (Shrier and Platt, 2008). Therefore, single exposure mixed effect models were additionally adjusted for the following characteristics: season (winter, summer), area of agricultural fields in 250 m radius around the households (in m^2^, continuous) (adjusted model 1), frequency of organic food consumption (<1 per month, 1–3 per month, 1 per week, 2–6 per week, daily), amount of fruit consumed in 3 days before sampling (number of pieces), amount of vegetable consumed in 3 days before sampling (number of pieces) (adjusted model 2). Variables adult smoking status (never smoker, former smoker, current smoker), household income (<25%, 25–50%, 50–75%, >75% of South-Moravian region average), adult education (primary, secondary, tertiary, university) were identified as redundant by DAG and were not included in the models to avoid over adjustment (see SI Fig. 1). To account for multiplicity (72 comparisons), false discovery rate (FDR) procedure was applied and false coverage-statement rate adjusted 95% confidence intervals (FCR-adjusted 95% CIs) were constructed (Benjamini and Yekutieli, 2005). Several sensitivity analyses were performed. First, we changed the urine dilution adjustment approach by constructing models of SG-corrected biomarkers of exposure and response instead of including SG as a covariate. Second, a 90% winsorizing transformation method was applied to reduce the effect of possible outliers. Third, we estimated effects using multiple exposure mixed effects model additionally adjusted for multiple urinary CUP metabolites.

## 3 Results

Demographic and behavioral characteristics are presented in Table 1. Median age of study participants was 41 and 9 years for adults and children, respectively. Among adults prevail females (65%) with boys as an offspring (57%). At the beginning of the study, the majority of participants (64% of adults and 89% of children) had their BMI in normal range. Most adults had a university education (78%) and were predominantly non-smokers (88%). Households were both in urban and agricultural areas with 34% of them lacking agricultural fields within a radius of 250 m around the household.

**Table 1:** Demographic characteristics of the study population at baseline.

Characteristic	
Age (years)	Median (Min – Max)
Parent	41 (31–54)
Child	9 (4–15)
Sex	Percent
Adults	
Female (%)	65
Male (%)	35
Children	
Girls (%)	43
Boys (%)	57
BMI	Percent
Adults	
Underweight or normal (<25) (%)	64
Overweight (25–30) (%)	31
Obese (>30) (%)	5
Children	
Underweight or normal (<25) (%)	89
Overweight (25–30) (%)	8
Obese (>30) (%)	3
Adult education	Percent
Primary and secondary (%)	6
Tertiary (%)	16
University (%)	78
Adult smoking status	Percent
Never smoker (%)	68
Former smoker (%)	20
Current smoker (%)	12
Household income^1^	Percent
1st quartile (%)	17
2nd quartile (%)	46
3rd quartile (%)	20
4th quartile (%)	17
Any agricultural area around the household^2^	Percent
No (%)	34
Yes (%)	66

1 Total household income (% of South-Moravian region average).

2 Presence of agricultural fields within a radius of 250 m around the household.

Descriptive statistics of SG adjusted and non-adjusted urinary levels of response biomarkers and CUP metabolites coupled with the LOQ, LOD and detection frequency for each measured compound are summarized in Table 2 and SI Tables 2–3. In our study, response biomarkers were present in all urine samples (n = 440). Out of CUP metabolites, the most frequently detected metabolite was TEB-OH, with a detection frequency varying from 94.6% to 99.1% across the seasons and subgroups, followed by 3-PBA (51.8%–88.2%), t/c-DCCA (50%-86.4), and TCPY (40.9%-87.3). Concentrations of all biomarkers (both response biomarkers and CUP metabolites) were higher in children than in adults in both seasons except of TEB-OH in the winter season. When comparing urinary biomarker levels in the winter and summer seasons, we observed a few significant differences (p < 0.05). Most of them were characterized by higher levels in winter: 8-OHdG, 5-mC, 3-PBA, t/c-DCCA, TCPY in children and 5-mC, 3-mA, t/c-DCCA, TCPY in adults. The only exception was the 7-mG biomarker in adults, which was detected in statistically significant higher concentrations in summer samples.

**Table 2:** Specific gravity adjusted urinary levels of CUP metabolites and biological response biomarkers with an overall detection frequency higher than 40%.

Biomarker (ng/mL)	LOD/LOQ	DF (%)	GM (GSD)	P95	DF (%)	GM (GSD)	P95
Adults							
		Winter season (n = 110)			Summer season (n = 110)		
8-OHdG	0.05/0.17	100	5.43 (1.42)	9.42	100	5.62 (1.44)	10.2
5-mC***	0.05/0.17	100	22.0 (1.62)	48.7	100	17.5 (1.72)	36.5
5-mdC	0.10/0.33	100	16.9 (1.49)	29.6	100	16.8 (1.58)	33.4
5-hmdC	0.05/0.17	100	1.73 (1.59)	3.90	100	1.71 (1.70)	3.977
7-mG***	1.00/3.33	100	1778 (1.85)	5881	100	2223 (1.65)	4962
3-mA***	0.10/0.33	100	9.20 (2.45)	36.1	100	7.29 (2.35)	29.7
3-PBA	0.04/0.14	51.8	0.121 (4.03)	0.905	51.8	0.123 (4.62)	1.18
t/c-DCCA*	0.03/0.11	60.9	0.300 (6.10)	3.16	50	0.195 (5.02)	1.77
TCPY***	0.03/0.09	87.3	2.29 (3.93)	7.37	40.9	0.243 (6.13)	3.07
TEB-OH	0.02/0.05	98.2	0.459 (2.45)	1.75	94.6	0.494 (2.98)	4.05
Children							
		Winter season (n = 110)			Summer season (n = 110)		
8-OHdG***	0.05/0.17	100	6.72 (1.38)	10.8	100	5.70 (1.45)	10.3
5-mC***	0.05/0.17	100	31.7 (1.63)	65.8	100	26.3 (1.85)	59.7
5-mdC	0.10/0.33	100	24.5 (1.54)	43.7	100	23.9 (1.49)	44.5
5-hmdC	0.05/0.17	100	2.74 (1.47)	6.00	100	2.71 (1.51)	6.08
7-mG	1.00/3.33	100	3377 (1.70)	7407	100	3390 (1.61)	6984
3-mA	0.10/0.33	100	11.4 (2.64)	54.3	100	9.18 (2.64)	42.3
3-PBA*	0.04/0.14	88.2	0.465 (3.65)	2.26	82.7	0.317 (3.72)	1.57
t/c-DCCA***	0.03/0.11	86.4	1.08 (5.25)	6.66	76.4	0.534 (4.14)	3.23
TCPY**	0.03/0.09	83.6	2.53 (5.37)	9.73	83.6	1.17 (4.67)	4.44
TEB-OH	0.02/0.05	99.1	0.459 (2.26)	1.77	97.3	0.558 (3.29)	9.86

Abbreviations: 8-OHdG: 8-hydroxydeoxyguanosine, 5-mC: 5-methylcytosine, 5-mdC: 5-Methyl-2′-deoxycytidine, 5-hmdC: 5-hydroxymethyl-2′-deoxycytidine, 7-mG: 7-methylguanine, 3-mA: 3-methyladenine, 3-PBA: 3-phenoxybenzoic acid, t/c-DCCA: trans/cis-3-(2,2-dichlorovinyl)-2,2-dimethylcyclopro-pane carboxylic acid, TCPY: 3,5,6-trichloro-2-pyridinol, TEBOH: hydroxy-1-tebuconazole.

DF = detection frequency.

GM = geometric mean.

GSD = geometric standard deviation.

P95 = 95th percentile.

*p < 0.05, **p < 0.01, ***p < 0.001 for significant difference in biomarker concentration between seasons, estimated from LME model with random intercepts for participant ID and specific household, adjusted for age, BMI, sex, specific gravity, agricultural area, fruit consumption, vegetable consumption, organic food consumption.

Correlation analysis among the response biomarkers across the seasons and subgroups separately (children in winter, children in summer, adults in winter, adults in summer) showed some statistically significant patterns. The highest Pearson correlation coefficients were observed between 5-mdC and 8-OHdG (r ranging from 0.37 to 0.55 among the seasons and subgroups, p < 0.0001) and between 5-mdC and 5-mC (r = 0.34–0.58, p < 0.001). Weaker correlations were observed between 5-mdC and 5-hmdC (r = 0.25–0.42, p < 0.01) and between 8-OHdG and 5-mC (r = 0.19–0.39, p < 0.05). The remaining correlations were observed only in some season and/or subgroup or were insignificant, showing no conclusive pattern (see SI Tables 4–7). Significant positive correlations were also found between some CUP metabolites and are published and discussed in detail elsewhere (Šulc et al., 2022).

Estimates of effects from LME models showed some robust associations across all diversly adjusted models and results are given in Table 3. Results are presented as a percentage change in response biomarker concentrations associated with inter-quartile range (IQR) change in the urinary concentration of a CUP metabolite. Both pyrethroid metabolites (3-PBA and t/c-DCCA) were associated with an increase in the concentration of oxidative stress biomarker 8-OHdG in the final fully adjusted model 2. The percentage change in 8-OHdG associated with IQR change was 10.2% (95% CI: 2.85, 18.1) in the case of 3-PBA and 11.6% (95% CI: 2.47, 21.5) in the case of t/c-DCCA. Furthermore, IQR change in t/c-DCCA concentration was also associated with 13.6% change (95% CI: 2.50, 25.8) in 5-mdC concentration in adjusted model 2. Similarly, higher concentrations of 5-mdC and 5-mC were also associated with chlorpyrifos metabolite TCPY (% change = 11.6%; 95% CI: 0.48, 23.9 and 14.6%; 95% CI: 1.58, 29.2, respectively). The only negative estimate (increase in CUP metabolite associated with a decrease in response biomarker) was found between TEB-OH and 3-mA (−15.1%; 95% CI: 23.6, −5.69) which is also the most robust effect observed across all models. Significant results of adjusted model 2 were similar to adjusted model 1 and base model. The only exception was decrease in strength of the association between TCPY and 5-mC which was observed when comparing base model and adjusted model 1. The remaining associations were not significant or conclusive. Significant results of the final fully adjusted model 2 were robust and observed also in sensitivity analysis. We observed a few increases/decreases in effect estimates, however still significant, when using SG-corrected biomarker levels, applying winsorizing and constructing multiple exposure mixed effects model additionally adjusted for multiple urinary CUP metabolites (SI Table 8).

**Table 3:** Percentage change and FCR-adjusted 95% confidence interval in urinary response biomarkers associated with IQR increase in urinary CUP metabolite concentrations.

	Base model a	Adjusted model 1 b	Adjusted model 2 c
	% change (95% CI)∗	% change (95% CI)∗	% change (95% CI)∗
8-OHdG			
3-PBA	**10.5 (3.27, 18.3)**	**9.82 (2.61, 17.5)**	**10.2 (2.85, 18.1)**
t/c-DCCA	**14.3 (5.26, 24.2)**	**12.7 (3.56, 22.7)**	**11.6 (2.47, 21.5)**
TCPY	**8.92 (0.80, 17.7)**	6.07 (−2.83, 15.8)	5.37 (−3.48, 15.0)
TEB-OH	3.09 (−2.06, 8.51)	3.53 (−1.62, 8.96)	3.94 (−1.3, 9.46)
5-mC			
3-PBA	−0.44 (−10.0, 10.1)	−2.48 (−11.5, 7.49)	−4.59 (−13.6, 5.37)
t/c-DCCA	10.7 (−1.97, 25.0)	4.82 (−7.12, 18.3)	4.65 (−7.3, 18.1)
TCPY	**24.6 (11.9, 38.7)**	**14.6 (1.61, 29.2)**	**14.6 (1.58, 29.2)**
TEB-OH	−1.19 (−8.18, 6.34)	−0.01 (−6.85, 7.32)	−0.39 (−7.31, 7.05)
5-mdC			
3-PBA	3.87 (−4.38, 12.8)	3.65 (−4.63, 12.6)	3.22 (−5.21, 12.4)
t/c-DCCA	**14.0 (3.29, 25.9)**	**14.0 (2.97, 26.2)**	**13.6 (2.50, 25.8)**
TCPY	**11.2 (1.39, 21.9)**	**12.5 (1.31, 24.8)**	**11.6 (0.48, 23.9)**
TEB-OH	0.58 (−5.36, 6.9)	0.74 (−5.25, 7.11)	0.90 (−5.20, 7.39)
5-hmdC			
3-PBA	4.41 (−4.25, 13.9)	4.14 (−4.50, 13.6)	5.02 (−3.92, 14.8)
t/c-DCCA	3.99 (−6.37, 15.5)	3.80 (−6.75, 15.5)	3.26 (−7.37, 15.1)
TCPY	7.76 (−2.20, 18.7)	8.34 (−2.91, 20.9)	8.74 (−2.64, 21.4)
TEB-OH	0.37 (−5.85, 6.99)	0.99 (−5.28, 7.68)	1.83 (−4.64, 8.73)
7-mG			
3-PBA	6.46 (−3.76, 17.8)	7.34 (−2.96, 18.7)	7.91 (−2.56, 19.5)
t/c-DCCA	3.40 (−8.43, 16.8)	6.22 (−6.13, 20.2)	5.18 (−7.08, 19.1)
TCPY	4.15 (−7.21, 16.9)	11.8 (−1.63, 27.1)	12.2 (−1.21, 27.4)
TEB-OH	6.94 (−0.71, 15.2)	6.69 (−0.94, 14.9)	6.75 (−0.96, 15.1)
3-mA			
3-PBA	7.86 (−6.57, 24.5)	5.52 (−8.43, 21.6)	5.92 (−8.40, 22.5)
t/c-DCCA	−5.91 (−21.0, 12.1)	−12.1 (−26.4, 4.88)	−13.4 (−27.5, 3.47)
TCPY	16.0 (−1.03, 36.0)	4.49 (−12.7, 25.0)	4.80 (−12.5, 25.5)
TEB-OH	**−16.1 (-24.4, -6.84)**	**−15.0 (-23.3, -5.78)**	**−15.1 (-23.6, -5.69)**

Abbreviations: 8-OHdG: 8-hydroxydeoxyguanosine, 5-mC: 5-methylcytosine, 5-mdC: 5-Methyl-2′-deoxycytidine, 5-hmdC: 5-hydroxymethyl-2′-deoxycytidine, 7-mG: 7-methylguanine, 3-mA: 3-methyladenine, 3-PBA: 3-phenoxybenzoic acid, t/c-DCCA: trans/cis-3-(2,2-dichlorovinyl)-2,2-dimethylcyclopro-pane carboxylic acid, TCPY: 3,5,6-trichloro-2-pyridinol, TEBOH: hydroxy-1-tebuconazoleEstimates from linear mixed effects models with random intercepts for participant ID and households (n = 440, 220 subjects). Levels of biomarkers were ln transformed.

a Base model, adjusted for age, BMI, sex, specific gravity.

b Adjusted model 1, adjusted for the same variables as Base model + season, agricultural area.

c Adjusted mode 2, adjusted for the same variables as Adjusted model 1 + fruit consumption, vegetable consumption, organic food consumption.

∗ 95% confidence intervals were false coverage-statement adjusted to account for multiple testingIQR 3-PBA = 0.560 ng/mL; IQR t/c-DCCA = 1.46 ng/mL; IQR TCPY = 4.15 ng/mL; IQR TEB-OH = 0.567 ng/mL.

## 4 Discussion

In the population of Czech adults and children from the CELSPAC-SPECIMEn cohort, we examined urinary levels of DNA methylation and oxidative stress biomarkers in repeatedly collected samples from the winter and summer seasons. Urinary levels of CUP metabolites were also examined and higher levels in children's urine were found in comparison to adult samples. In adults, urinary CUP levels were often similar among seasons, in children higher in winter. Detailed discussion of the results is provided elsewhere (Šulc et al., 2022). We observed that levels of all response biomarkers were higher in children's urine samples in both seasons. It is reasonable to expect, as age-related DNA methylation patterns were reported to have regulatory roles on gene activity and developmental processes. Hence the increased levels of response biomarkers in children are mainly caused due to extensive development during childhood (Gervin et al., 2016). The shortened period for DNA repair and the multiple changes that are occurring within DNA, together with different toxicokinetics of many environmental pollutants, could also lead to increased susceptibility and vulnerability to environmental pollutants in children, subsequently leading to increased DNA alterations (Bearer, 1995).

The children's 8-OHdG levels found in this study were slightly lower than those reported among children from Japan (Ait Bamai et al., 2019), Uruguay (Kordas et al., 2018) and the US and Canada (Jacobson et al., 2020). However, similar levels to our study were observed in Chinese young children (Wei et al., 2022). Such slight deviations in urinary 8-OHdG levels could be explained by the usage of different methods with higher levels reported for immunological techniques compared to chemical methods (Graille et al., 2020). As the immunochemical enzyme-linked immunosorbent assays (ELISA) has several analytical limitations, chromatographic methods are considered to be the gold standard (Bláhová et al., 2023 – manuscript submitted). The same is true when comparing the adult 8-OHdG levels, which are mostly in line with previous studies when considering chemical analytical techniques and slightly lower compared to immunological ones (Graille et al., 2020).

Data on methylated DNA bases in the urine of general population are sparse so far. Although their usage could be very useful. By measuring urinary levels of these biomarkers, changes of the DNA methylation status in the whole body could be assessed and the DNA demethylation mechanisms could be investigated in vivo (Hu et al., 2012). In addition, such a non-invasive measurement could serve as a useful biomarker of exposure to methylating agents or other xenobiotics (Hu et al., 2011; Lovreglio et al., 2020) and as a promising early biomarker of several disorders (Pan et al., 2016; Onishi et al., 2019). There are few studies in occupationally exposed populations or in population sub-groups. In the occupational study of Lovreglio et al. (2020), control group (n = 93) from Italy showed the same levels of 7-mG and 5-mC, but lower levels of 5-mdC (median 2.77 μg/g crea.). Contrary, urinary levels of 5-mdC and 5-hmdC of male partners (n = 562) of subfertile couples from China were in line with our findings (Pan et al., 2016). Higher urinary levels of 5-mC, 7-mG, and lower levels of 5-mdC, 3-mA were reported in the study of 376 healthy male subjects from Taiwan, but still within the same order of magnitude (Hu et al., 2012). These slight discrepancies might be linked to the study population, as it has already been proposed that global DNA methylation patterns differ with subject and lifestyle characteristics, such as age, gender, alcohol drinking (Zhu et al., 2012), or subject health status (Robertson, 2005). Considering the health status, in these specific cases, urinary levels of 5-mdC significantly differed with progression of chronic kidney disease (Onishi et al., 2019) and those of 3-mA and 7 mG between Parkinson's Disease and Parkinsonian Syndromes Patients compared to control group (Gątarek et al., 2020). To the best of our knowledge, there is no previous study in children population investigating urinary biomarkers of potential nucleic acid methylation.

The repeated sampling design of our study allows us to do the first comparison of levels of oxidative stress and nucleic acid methylation biomarkers of general population in two different seasons (winter vs. summer). Increased concentrations could be seen particularly in urine samples from the winter season. There are several possible explanations for the observed pattern. Many seasonal factors such as temperature and light could affect transcriptional mechanisms via DNA methylation (Alvarado et al., 2014). The total antioxidant capacity of a human system was also proven to vary seasonally with significantly greater capacity in the summer season (Morera-Fumero et al., 2018). This could be the consequence of different dietary patterns between summer and winter seasons, especially considering fruit and vegetable consumption as a principal source of an antioxidative potential in a diet (Capita and Alonso-Calleja, 2005; Człapka-Matyasik and Ast, 2014). Last, but not least, exposure to some environmental chemicals is expected to be dissimilar between seasons due to distinct behavioral habits. As demonstrated in the case of exposure to CUPs in the present study and discussed in detail previously (Šulc et al., 2022) or in the case of multiple volatile organic compounds due to increased time spent indoors and reduced ventilation during winter season (Paciência et al., 2016).

The significant, robust (across both seasons in both adults and children) correlations were found among some biomarkers. The positive correlations of urinary 5-mdC with its oxidized form 5-hmdC and nucleobase 5-mC are consistent among other studies (Hu et al., 2012; Pan et al., 2016) because all of them may be urinary products of methylation within the cytosine nucleotide. Urinary 5-mdC was, along with 5-mC, further correlated with urinary biomarker of oxidative stress 8-OHdG. This suggests that increased oxidative stress may exhaust antioxidant activity which is biochemically linked to biosynthesis of S-adenosylmethionine (SAM), an important methyl donor for DNA methylation and thus induce increased methylation (de Prins et al., 2013). Whereas methylation of the C-5 position of cytosine is predominantly catalyzed enzymatically, 3-mA and 7-mG are products of nonenzymatic DNA methylation. Therefore, no conclusive pattern was found when considering correlations with 7-mG and 3-mA biomarkers. However, Hu et al., 2012 found significant associations of 5-mC with 3-mA and 7-mG biomarkers, confirming that SAM, as a methyl donor for enzymatic methylation, may play an important role in nonenzymatic methylation as well.

The effects of CUPs on oxidative stress and DNA methylation in Czech adults and children were examined using LME model. An important observation in our study was the positive association of pyrethroid metabolites (3-PBA and t/c-DCCA) with the urinary level of the oxidative stress biomarker. The effects of pyrethroid exposure on oxidative DNA damage have already been explored by animal exposure studies and proposed as one of the mechanisms linked to pesticide-induced chronic diseases (Banerjee et al., 2001). In such cases, increased levels of oxidative stress biomarkers and/or enzymes and decreased levels of antioxidants suggest the involvement of pyrethroid pesticides in oxidative stress generation (Kale et al., 1999; Aouey et al., 2017). Similar changes in antioxidant enzymes and biomarkers of oxidative stress were observed in studies from occupational setting (Sharma et al., 2013; Zepeda-Arce et al., 2017). Nevertheless, results are inconclusive despite the fact that agricultural workers may by constantly exposed to remarkably higher levels of pyrethroids. In general population, studies of CUPs in relation to oxidative stress biomarkers are limited. The only general population study among primary school children in Cyprus brings consistent results with our study (Makris et al., 2022). Using creatinine-adjusted biomarkers of exposure and effect, urinary levels of 8-OHdG were significantly associated with 3-PBA metabolite (β = 0.19, 95% CI: 0.02, 0.37) but at the edge of significance with t/c-DCCA (β = 0.12, 95% CI: 0.02, 0.27 and β = 0.12, 95% CI: 0.02, 0.25 for cis- and trans-respectively). A significant association was also observed for the chlorpyrifos metabolite TCPY (β = 0.42, 95% CI: 0.16, 0.68) which is in agreement with other studies exploring effects of organophosphate pesticides on oxidative stress. Increased urinary levels of 8-OHdG were reported on the first day after chlorpyrifos spraying in the case of farmers (Wang et al., 2016) and decreased levels of glutathione, which is part of an antioxidant system, were found among children in the agricultural community compared to the urban community (Sapbamrer et al., 2020). On the contrary, urinary levels of TCPY were not associated with urinary levels of 8-OHdG in our study which may be related to relatively strict parameters of our models (multiple adjustment variables, multiple testing correction) compared with above mentioned studies.

In addition to indicating effects of pyrethroids on oxidative stress, our results suggest that CUP exposure might induce changes in DNA methylation patterns (either hyper- or hypo-methylation). The proposed mechanism of environmental chemicals action consists mainly of an altered function of methyl donor SAM and enzyme DNA methyltransferases, which catalyzes the transfer of the methyl group (Ruiz-Hernandez et al., 2015). This is supported by few epidemiological studies which have reported altered DNA methylation levels of specific gene promoters in response to pesticide mixture exposure (Rusiecki et al., 2017; Declerck et al., 2017, 2017van der Plaat et al., 2018; Benitez-Trinidad et al., 2018). Considering global DNA methylation changes, Benedetti et al. (2018) carried out the study on soybean farmers who were actively engaged in the preparation and application of the complex mixture of pesticides. The results showed a significant difference in the percentage of global DNA methylation in individuals exposed to pesticides compared to the control group. Whereas most of the studies are usually conducted in an occupational setting and are not focused on specific CUPs, general population evidence is limited. In the present investigation, the associations between urinary biomarkers of DNA methylation and CUP exposure were studied for the first time. The robust associations of 5-mC and its deoxynucleoside 5-mdC with chlorpyrifos and pyrethroid metabolites were observed across all models. Nevertheless, proportion of imputed data in some study strata (particularly TCPY in adults in summer) was high which could potentially lead to bias. Furthermore, higher urinary levels of TEB-OH were importantly associated with lower levels of urinary 3-mA indicating a potential role of tebuconazole in hypomethylation or decreased demethylation of DNA. Tebuconazole is often used in a mixture with prothioconazole which are triazole and triazole-thionine based fungicides, respectively. Both of them are used to control fungal plant diseases of major crops like cereals and canola or for seed treatment (Jørgensen and Heick, 2021) and are the most frequently used azole pesticides in the Czech Republic (CISTA, 2022). The possible mechanism of their effect may consist of alkyladenine-DNA glycosylase (AAG) inhibition. It is well known that excision repair of 3-mA in DNA is initiated by AAG enzyme with subsequential generation of an apurinic/apyrimidinic site (Fu et al., 2012). Simultaneously, potential inhibition of AAG by triazole-thione-based compounds is proposed (Al and Ba, 2017) which could lead to decreased demethylation processes and therefore to increased methylation of adenine in DNA and decreased presence of 3-mA in urine.

When comparing effect estimates from all diversly adjusted models, only notable change was observed in the case of association between chlorpyrifos metabolite and 5-mC. Decrease of the effect estimate is expected to be caused by season adjustment as significant seasonal differences were observed in both biomarkers (see Table 2). Robustness of the results when replicating LME models in sensitivity analyses showed that associations are not influenced by method used for urine dilution adjustment, by outliers neither by multiple urinary CUP metabolites.

There are some limitations to this study. Firstly, as measurement of urinary response biomarkers reflects the results of DNA repair processes in the “whole body”, we are not able to interpret the results as an epigenetic change within regional or individual genes (e.g. DNA hypermethylation of tumor suppressor genes), which could be useful when associating with specific health outcomes. Secondly, both exposure and response biomarkers were measured in a first morning void urine samples and thus may not be fully representative of a long-term temporal variability in the given season. Thirdly, considering environmental complexity, observed associations could be confounded by other unmeasured environmental chemical exposures or other factors, despite the fact that potential confounding variables were carefully selected. Finally, the data on CUP biomarkers were imputed. Although imputation is common scientific practice, imputation cutoff (40%) could potentially influence the results. This should be considered especially in the case of TCPY. As we are primarily interested in the detection of possible new associations, rather than confirming with certainty hypothesized associations, we accepted the risk of potential false-positive results. On the other hand, these limitations are countered by a number of strengths. Mainly, repeated measurements in winter and summer in both adults and children allowed us to cover variability in both exposure and response biomarkers across the seasons and population subgroups. Furthermore, the wide scope of response biomarkers, assessed using a more precise mass spectrometry method, enhanced our ability to reveal more possible effects in the human body.

## 5 Conclusion

In conclusion, to the best of our knowledge, this is the first study to measure and describe biomarkers of DNA methylation and oxidative stress in urine samples of Czech adult population and the first in children overall. In addition, it is the first epidemiological study to assess the associations of urinary biomarkers of response with CUP exposure. We observed significant, robust associations across all assessed models. Pyrethroid metabolites were associated with higher levels of both oxidative stress and DNA methylation biomarkers. Moreover, urinary levels of the chlorpyrifos metabolite were also associated with urinary products of methylation within the cytosine nucleotide. Finally, the most robust, negative association was observed between the tebuconazole metabolite and 3-methyladenine indicating a possible role of azole pesticides in demethylation processes. These findings suggest an urgent need to extend the range of analyzed environmental chemicals such as azole pesticides (for instance prothioconazole) in human biomonitoring studies to responsibly evaluate associated health risks. In addition, observed associations warrant further large-scale research of these biomarkers and environmental pollutants including CUPs.

## Credit author statement

**Tomáš Janoš:** Conceptualization, Methodology, Formal analysis, Investigation, Data curation, Writing – original draft, Visualization, **Ilse Ottenbros:** Conceptualization, Methodology, Writing – review & editing, **Lucie Bláhová:** Methodology, Validation, Investigation, Writing – review & editing, **Petr Šenk:** Methodology, Validation, Investigation, Writing – review & editing, **Libor Šulc:** Methodology, Writing – review & editing, **Nina Pálešová:** Investigation, Writing – review & editing, **Jessica Sheardová:** Writing – review & editing, Visualization, **Jelle Vlaanderen:** Conceptualization, Methodology, Resources, Writing – review & editing, Supervision, **Pavel Čupr:** Conceptualization, Methodology, Resources, Writing – review & editing, Supervision, Project administration, Funding acquisition.

## Declaration of competing interest

The authors declare that they have no known competing financial interests or personal relationships that could have appeared to influence the work reported in this paper.

## Data Availability

Data will be made available on request.
