# Supplementary material for: Effects of pesticide exposure on oxidative stress and DNA methylation urinary biomarkers in Czech adults and children from the CELSPAC-SPECIMEn cohort
Source: Environ Res. 2023 Apr 1;222:115368. doi: 10.1016/j.envres.2023.115368 (PMC10009299; doi:10.1016/j.envres.2023.115368)
Supplement: Multimedia component 1 [file mmc1.docx]

**Supplementary information for**

**“Effects of pesticide exposure on oxidative stress and DNA methylation urinary biomarkers in Czech adults and children from the CELSPAC-SPECIMEn cohort”**

**Authors:**

Tomáš Janoš^1^, Ilse Ottenbros^2,3^, Lucie Bláhová^1^, Petr Šenk^1^, Libor Šulc^1^, Nina Pálešová^1^, Jessica Sheardová^1^, Jelle Vlaanderen^2^, Pavel Čupr^1^*

**Affiliations:**

^1^ RECETOX, Faculty of Science, Masaryk University, Kotlarska 2, Brno, Czech Republic

^2^ Institute for Risk Assessment Sciences, Utrecht University, Utrecht, the Netherlands

^3^ Center for Sustainability, Environment and Health, National Institute for Public Health and the Environment (RIVM), Bilthoven, Netherlands

*****Corresponding author: Pavel Čupr, RECETOX Centre, Faculty of Science, Masaryk University, Kamenice 753/5, pavilion A29, 625 00 Brno, Czech Republic, Phone number: +420 549 493 511, E-mail: [pavel.cupr@recetox.muni.cz](mailto:pavel.cupr@recetox.muni.cz)

**SI Table 1** Mass spectrometer and validation parameters.

| **Biomarker** | **CAS** | **Retention time (min)** | **LOD µg/L** | **Recovery (%)^a^** | **MRM transition** | **Cone (V)** | **Collision (V)** |
| --- | --- | --- | --- | --- | --- | --- | --- |
| 5-mdC-d3 | 554-01-8 | 2.4 |  |  | **245.1 > 129.1**  245.1>112.2 | 35 | 15  35 |
| 3-mA-d3 |  | 1.9 |  |  | **154.2 > 109.3**  154.2 > 127.1  154.2 > 137.3 | 35 | 20  20  20 |
| 15N5-8-OHdG |  | 3.2 |  |  | **289.1 > 173.1**  289.1 > 114.1 | 30 | 14  28 |
| 5-mC | 554-01-8 | 2.0 | 0.05 | 107±8 | **126.2>109.2**  126.2>83.2  126.2>56.2 | 35 | 20  21  24 |
| 5-mdC | 838-07-3 | 2.4 | 0.1 | 109±12 | **242.1>126.2**  242.1>109.2 | 30 | 14  35 |
| 5-hmdC | 7226-77-9 | 1.9 | 0.05 | 107±15 | **258.2>142.2**  258.2>81.1  258.2>124.1 | 20 | 10  30  20 |
| 3-mA | 5142-23-4 | 1.9 | 0.1 | 115±23 | **150.2>133.1**  150.2>109.1  150.2>123.1 | 35 | 18  19  20 |
| 7-mG | 578-76-7 | 2.3 | 1 | 116±20 | **166.2>79.1**  166.2>124.1  166.2>69.2 | 35  37  30 | 25  20  30 |
| 8-OHdG | 88847-89-6 | 3.2 | 0.05 | 99±6 | **284.1 > 168.1**  284.1 > 140.1 | 30 | 14  28 |

^a^ mean recovery and RSD (%); spike 5, 50, 400 µg/L in 0.1% FA in triplicates

quantification ions are marked with **bold**

**SI Table 2** Descriptive statistic of specific gravity corrected levels of biological response biomarkers in human urine.

| Biomarker (ng/mL) |  |  |  | Percentiles |  |  |  |
| --- | --- | --- | --- | --- | --- | --- | --- |
|  | 5 | 10 | 25 | 50 | 75 | 90 | 95 |
|  |  |  |  | Adults |  |  |  |
| 8-OHdG | 1.568 | 3.091 | 3.652 | 4.335 | 5.415 | 7.138 | 8.812 |
| 5-mC | 1.354 | 8.882 | 10.784 | 14.442 | 19.092 | 26.341 | 35.030 |
| 5-mdC | 4.134 | 8.453 | 9.930 | 12.714 | 16.961 | 22.635 | 28.841 |
| 5-hmdC | 0.404 | 0.843 | 0.988 | 1.240 | 1.643 | 2.388 | 2.951 |
| 7-mG | 370.119 | 866.584 | 978.778 | 1421.848 | 2011.615 | 2609.544 | 4023.126 |
| 3-mA | 0.963 | 1.951 | 2.599 | 4.301 | 8.390 | 14.563 | 27.256 |
|  |  |  |  | Children |  |  |  |
| 8-OHdG | 1.953 | 3.581 | 4.086 | 4.892 | 6.189 | 8.043 | 9.520 |
| 5-mC | 1.221 | 12.192 | 15.664 | 21.973 | 29.810 | 41.047 | 52.204 |
| 5-mdC | 4.110 | 12.786 | 14.601 | 18.466 | 24.517 | 32.381 | 40.161 |
| 5-hmdC | 1.118 | 1.552 | 1.678 | 2.067 | 2.584 | 3.492 | 4.694 |
| 7-mG | 584.504 | 1429.589 | 1768.177 | 2495.110 | 3507.206 | 4786.016 | 6374.581 |
| 3-mA | 0.775 | 1.770 | 2.827 | 5.724 | 10.574 | 20.755 | 36.867 |

**SI Table 3** Descriptive statistic of non-corrected levels of biological response biomarkers in human urine.

| Biomarker (ng/mL) |  |  |  | Percentiles |  |  |  |
| --- | --- | --- | --- | --- | --- | --- | --- |
|  | 5 | 10 | 25 | 50 | 75 | 90 | 95 |
|  |  |  |  | Adults |  |  |  |
| 8-OHdG | 1.121 | 1.993 | 2.344 | 3.352 | 4.976 | 7.017 | 11.508 |
| 5-mC | 2.150 | 6.322 | 7.724 | 11.242 | 16.998 | 27.066 | 43.615 |
| 5-mdC | 2.621 | 5.318 | 6.312 | 9.524 | 15.319 | 24.917 | 36.301 |
| 5-hmdC | 0.240 | 0.498 | 0.623 | 0.972 | 1.583 | 2.498 | 3.837 |
| 7-mG | 382.860 | 647.503 | 796.435 | 1192.555 | 1832.717 | 2624.415 | 3919.038 |
| 3-mA | 1.260 | 2.042 | 2.791 | 4.385 | 7.351 | 12.985 | 20.035 |
|  |  |  |  | Children |  |  |  |
| 8-OHdG | 1.441 | 2.474 | 3.212 | 4.378 | 6.319 | 8.868 | 10.666 |
| 5-mC | 1.523 | 10.016 | 12.613 | 18.193 | 29.298 | 42.457 | 57.237 |
| 5-mdC | 3.635 | 9.682 | 12.634 | 17.000 | 24.884 | 33.633 | 42.272 |
| 5-hmdC | 0.474 | 1.149 | 1.382 | 1.881 | 2.570 | 3.592 | 5.250 |
| 7-mG | 684.491 | 1098.193 | 1391.330 | 2235.783 | 3422.365 | 5165.194 | 7493.054 |
| 3-mA | 0.631 | 2.302 | 2.898 | 6.303 | 10.905 | 16.461 | 29.262 |

**SI Table 4** Correlation analysis (Pearson correlation coefficient (r) and respective p-value) among the response biomarkers in children, winter season (n=110).

| r | 8-OHdG | 5-mC | 5-hmdC | 5-mdC | 7-mG | 3-mA |
| --- | --- | --- | --- | --- | --- | --- |
| 8-OHdG |  | 0.386 | 0.235 | 0.368 | 0.130 | 0.068 |
| 5-mC |  |  | 0.321 | 0.551 | -0.046 | -0.044 |
| 5-hmdC |  |  |  | 0.355 | -0.218 | -0.023 |
| 5-mdC |  |  |  |  | -0.174 | 0.010 |
| 7-mG |  |  |  |  |  | -0.187 |
| 3-mA |  |  |  |  |  |  |
| p-value | 8-OHdG | 5-mC | 5-hmdC | 5-mdC | 7-mG | 3-mA |
| 8-OHdG |  | 0.00003 | 0.01355 | 0.00008 | 0.17425 | 0.47859 |
| 5-mC |  |  | 0.00062 | 0.00000 | 0.63066 | 0.64548 |
| 5-hmdC |  |  |  | 0.00014 | 0.02187 | 0.80818 |
| 5-mdC |  |  |  |  | 0.06985 | 0.91725 |
| 7-mG |  |  |  |  |  | 0.05001 |
| 3-mA |  |  |  |  |  |  |

**SI Table 5** Correlation analysis (Pearson correlation coefficient (r) and respective p-value) among the response biomarkers in children, summer season (n=110).

| r | 8-OHdG | 5-mC | 5-hmdC | 5-mdC | 7-mG | 3-mA |
| --- | --- | --- | --- | --- | --- | --- |
| 8-OHdG |  | 0.362 | 0.224 | 0.394 | 0.316 | 0.138 |
| 5-mC |  |  | 0.369 | 0.583 | 0.054 | 0.135 |
| 5-hmdC |  |  |  | 0.417 | 0.018 | -0.096 |
| 5-mdC |  |  |  |  | -0.063 | 0.085 |
| 7-mG |  |  |  |  |  | -0.190 |
| 3-mA |  |  |  |  |  |  |
| p-value | 8-OHdG | 5-mC | 5-hmdC | 5-mdC | 7-mG | 3-mA |
| 8-OHdG |  | 0.00010 | 0.01872 | 0.00002 | 0.00078 | 0.14939 |
| 5-mC |  |  | 0.00007 | 0.00000 | 0.57847 | 0.15823 |
| 5-hmdC |  |  |  | 0.00001 | 0.84978 | 0.31674 |
| 5-mdC |  |  |  |  | 0.51305 | 0.37969 |
| 7-mG |  |  |  |  |  | 0.04664 |
| 3-mA |  |  |  |  |  |  |

**SI Table 6** Correlation analysis (Pearson correlation coefficient (r) and respective p-value) among the response biomarkers in adults, winter season (n=110).

| r | 8-OHdG | 5-mC | 5-hmdC | 5-mdC | 7-mG | 3-mA |
| --- | --- | --- | --- | --- | --- | --- |
| 8-OHdG |  | 0.266 | 0.210 | 0.551 | 0.012 | -0.023 |
| 5-mC |  |  | 0.278 | 0.552 | -0.048 | -0.070 |
| 5-hmdC |  |  |  | 0.346 | -0.076 | -0.187 |
| 5-mdC |  |  |  |  | -0.260 | -0.153 |
| 7-mG |  |  |  |  |  | 0.287 |
| 3-mA |  |  |  |  |  |  |
| p-value | 8-OHdG | 5-mC | 5-hmdC | 5-mdC | 7-mG | 3-mA |
| 8-OHdG |  | 0.00495 | 0.02740 | 0.00000 | 0.90003 | 0.81192 |
| 5-mC |  |  | 0.00323 | 0.00000 | 0.61934 | 0.46878 |
| 5-hmdC |  |  |  | 0.00021 | 0.43180 | 0.05003 |
| 5-mdC |  |  |  |  | 0.00600 | 0.11093 |
| 7-mG |  |  |  |  |  | 0.00233 |
| 3-mA |  |  |  |  |  |  |

**SI Table 7** Correlation analysis (Pearson correlation coefficient (r) and respective p-value) among the response biomarkers in adults, summer season (n=110).

| r | 8-OHdG | 5-mC | 5-hmdC | 5-mdC | 7-mG | 3-mA |
| --- | --- | --- | --- | --- | --- | --- |
| 8-OHdG |  | 0.191 | 0.099 | 0.491 | 0.121 | 0.052 |
| 5-mC |  |  | 0.033 | 0.336 | -0.015 | 0.075 |
| 5-hmdC |  |  |  | 0.254 | -0.113 | -0.067 |
| 5-mdC |  |  |  |  | -0.297 | -0.174 |
| 7-mG |  |  |  |  |  | 0.164 |
| 3-mA |  |  |  |  |  |  |
| p-value | 8-OHdG | 5-mC | 5-hmdC | 5-mdC | 7-mG | 3-mA |
| 8-OHdG |  | 0.04561 | 0.30383 | 0.00000 | 0.20982 | 0.59029 |
| 5-mC |  |  | 0.72965 | 0.00033 | 0.87987 | 0.43917 |
| 5-hmdC |  |  |  | 0.00740 | 0.23865 | 0.48795 |
| 5-mdC |  |  |  |  | 0.00162 | 0.06913 |
| 7-mG |  |  |  |  |  | 0.08759 |
| 3-mA |  |  |  |  |  |  |

**SI Table 8** Sensitivity analysis - percentage change and 95% confidence interval in urinary response biomarkers associated with IQR increase in urinary CUP metabolite concentrations derived from LME model with emphasis on robust associations from main analysis.

|  | Specific gravity-corrected variables^1^ | Winsorizing^2^ | Multiple model - Multiple exposure mixed effect model^3^ |
| --- | --- | --- | --- |
|  | % change (95% CI) | % change (95% CI) | % change (95% CI) |
| 8-OHdG ~ 3-PBA | 8.64 (3.95, 13.54) | 11.17 (5.17, 17.51) | 7.77 (1.76, 14.14) |
| 8-OHdG ~ t/c-DCCA | 8.26 (2.85, 13.96) | 11.36 (4.16, 19.05) | 7.43 (0.04, 15.36) |
| 5-mC ~ TCPY | 10.49 (2.54, 19.04) | 11.99 (2.51, 22.34) | 14.84 (4.37, 26.35) |
| 5-mdC ~ t/c-DCCA | 10.34 (3.69, 17.41) | 14.94 (6.33, 24.24) | 13.23 (3.94, 23.36) |
| 5-mdC ~ TCPY | 8.36 (1.59, 15.58) | 9.36 (0.88, 18.56) | 10.79 (1.97, 20.37) |
| 3-mA ~ TEB-OH | -16.87 (-23.85, -9.25) | -19.25 (-26.41, -11.39) | -14.8 (-21.72, -7.27) |

Estimates from linear mixed effects models with random intercepts for participant ID and households (n=440, 220 subjects). Levels of biomarkers were ln transformed.

^1^Adjusted for age, BMI, sex, season, agricultural area, fruit consumption, vegetable consumption, organic food consumption

^2^Adjusted for age, BMI, sex, season, specific gravity, agricultural area, fruit consumption, vegetable consumption, organic food consumption

^3^Adjusted for age, BMI, sex, season, specific gravity, agricultural area, fruit consumption, vegetable consumption, organic food consumption, multiple urinary CUP metabolites

Abbreviations: 8-OHdG: 8-hydroxydeoxyguanosine, 5-mC: 5-methylcytosine, 5-mdC: 5-Methyl-2′-deoxycytidine, 5-hmdC: 5-hydroxymethyl-2’-deoxycytidine, 7-mG: 7-methylguanine, 3-mA: 3-methyladenine, 3-PBA: 3-phenoxybenzoic acid, t/c.DCCA: trans/cis-3-(2,2-dichlorovinyl)-2,2-dimethylcyclopro-pane carboxylic acid, TCPY: 3,5,6-trichloro-2- pyridinol, TEBOH: hydroxy-1-tebuconazole


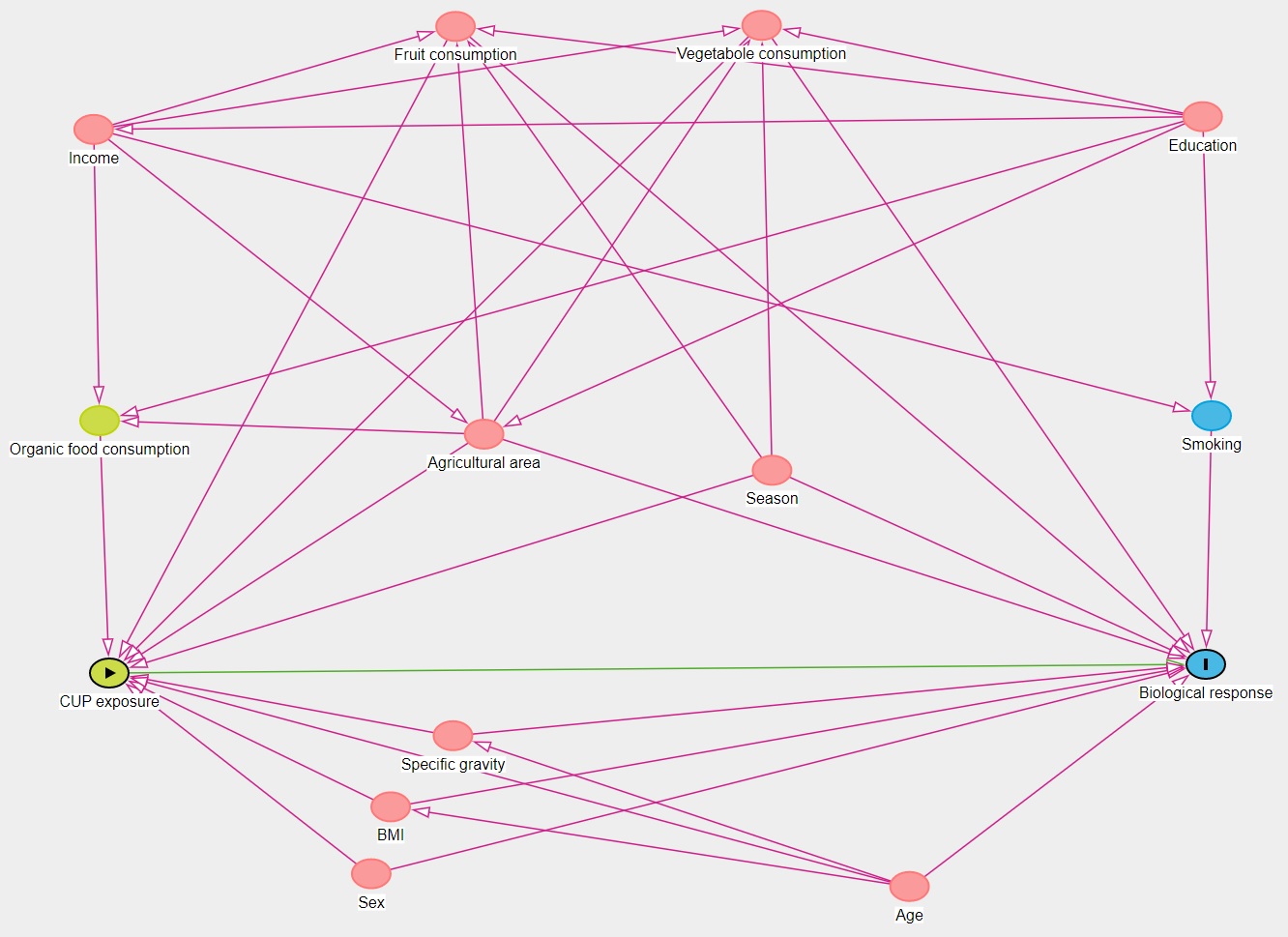


**SI Figure 1** Directed acyclic graph to study the associations between exposure to CUPs and biological response biomarkers.

Minimal sufficient adjustment sets for estimating the total effect of CUP exposure on Biological response: Age, Agricultural area, BMI, Fruit consumption, Organic food consumption, Season, Sex, Specific gravity, Vegetable consumption (Textor et al., 2016).

**References:**

Johannes Textor, Benito van der Zander, Mark K. Gilthorpe, Maciej Liskiewicz, George T.H. Ellison. Robust causal inference using directed acyclic graphs: the R package 'dagitty'. International Journal of Epidemiology 45(6):1887-1894, 2016
